# Metformin Protects Cardiovascular Health in People With Diabetes

**DOI:** 10.3389/fcvm.2022.949113

**Published:** 2022-07-12

**Authors:** Chong Chen, Shiqi Yuan, Xuenuo Zhao, Mengmeng Qiao, Shuna Li, Ningxia He, Liying Huang, Jun Lyu

**Affiliations:** ^1^Department of Clinical Research, The First Affiliated Hospital of Jinan University, Guangzhou, China; ^2^School of Public Health, Shannxi University of Chinese Medicine, Xianyang, China; ^3^Department of Neurology, The First Affiliated Hospital of Jinan University, Guangzhou, China; ^4^Qingdao University School of Public Health, Qingdao, China; ^5^Guangdong Provincial Key Laboratory of Traditional Chinese Medicine Informatization, Guangzhou, China

**Keywords:** metformin, cardiovascular health, logistic regression, NHANES, subgroup analysis

## Abstract

**Background:**

Metformin is the most commonly used drug for patients with diabetes, but there is still some controversy about whether it has a protective effect on cardiovascular health. We therefore used the National Health and Nutritional Examination Survey (NHANES) database to analyze the impact of metformin use on cardiovascular health in patients with diabetes.

**Methods:**

We extracted the demographic data and laboratory test results of all people with diabetes in the NHANES database from January 2017 to March 2020. The outcomes were seven indicators of cardiovascular health from the American Heart Association, each was scored as 0, 1, and 2 to represent poor, moderate, and ideal health statuses, respectively. The scores for the indicators (excluding diet and glycemic status) were summed, and the sum score was then considered to indicate unhealthy (0–5) or healthy (>5). Multivariate logistic regression analysis was used, and subgroup analyses were performed by age, alcohol consumption, education, and marital status.

**Results:**

This study included 1,356 patients with diabetes, among which 606 were taking metformin. After adjusting for all included variables, oral metformin in patients with diabetes had a protective effect on the cardiovascular health of patients (OR = 0.724, 95% CI = 0.573–0.913, *P* = 0.007). Subgroup analysis indicated that metformin protects the cardiovascular health of people with diabetes more clearly in those who are young (OR = 0.655, 95% CI = 0.481–0.892, *P* = 0.007), married (OR = 0.633, 95% CI = 0.463–0.863, *P* = 0.003), and drink alcohol (OR = 0.742, 95% CI = 0.581–0.946, *P* = 0.016).

**Conclusion:**

This study found that metformin has a protective effect on the cardiovascular health of patients with diabetes. The study findings support the general applicability of metformin.

## Background

Diabetes is a chronic disease caused by insufficient insulin secretion or by insulin utilization dysfunction in the body. As the course of the disease prolongs, long-term hyperglycemia toxicity will have adverse effects on other tissues and organs, thereby causing corresponding complications. Blood vessels are important target organs of diabetes, which can lead to cardiovascular disease if damaged. It is one of the ten most common causes of death worldwide, and in 2017 about 425 million people had diabetes worldwide (1, 2), thereby seriously affecting human health.

Metformin is currently the first-choice antiglycemic drug for patients with diabetes (3). Its mechanism of action is mostly to restore adenylyl cyclase inhibition using insulin through the G proteins of the liver cell membrane, reduce hepatic gluconeogenesis and hepatic glucose output, promote anaerobic glycolysis, increase the uptake and utilization of glucose by tissues such as skeletal muscle, inhibit or delay glucose absorption in the gastrointestinal tract, and improve glucose metabolism. It has also been recently found that metformin can increase the GLP-1 concentration in blood, and increase insulin sensitivity. Studies of metformin have also recently found that it can increase fibrinolysis and improve blood lipid concentrations, and plays a pivotal role in patients with diabetes.

Cardiovascular mortality has declined recently. However, research indicates that in 2019, the number of cardiovascular deaths worldwide reached 18.6 million, and it is still the most common cause of death worldwide (4). Studies have found that diabetes is an important risk factor for cardiovascular disease and death (5). It is therefore particularly important to pay attention to the cardiovascular health of patients with diabetes.

The cardiovascular effects of metformin in patients with diabetes have been controversial (6, 7). We therefore extracted the medication status of patients with diabetes from the National Health and Nutritional Examination Survey (NHANES) database to study the impact of the metformin on the patient's cardiovascular health, with the aim of improving to the cardiovascular health among people with diabetes.

## Methods

### Data Sources

The NHANES is a nutritional status study program of all populations in the United States that combines interviews and physical examinations (8). The survey annually examines a nationally representative sample of 5,000 people, with interviews covering demographics, socioeconomics, diet, and health-related questions, including medical, dental, and physiological measurements, as well as laboratory measurements by medical professionals (9). Importantly, the NHANES project information and its survey data are updated on the website in a timely manner and are freely available to the public.

### Research Variables

This study included subject data from March 2017 to March 2020, in which patients with diabetes were identified based on questionnaires and laboratory tests that included (1) providing information on the presence of diabetes in the questionnaire, and (2) a glycated hemoglobin level of 6.5% and fasting blood glucose at ≥126 mg/dL (10). The main parameters included in the study were age, sex, race, marital status, education, BMI, physical activity, smoking and alcohol statuses, metformin use, and related laboratory indicators.

**Figure 1:**
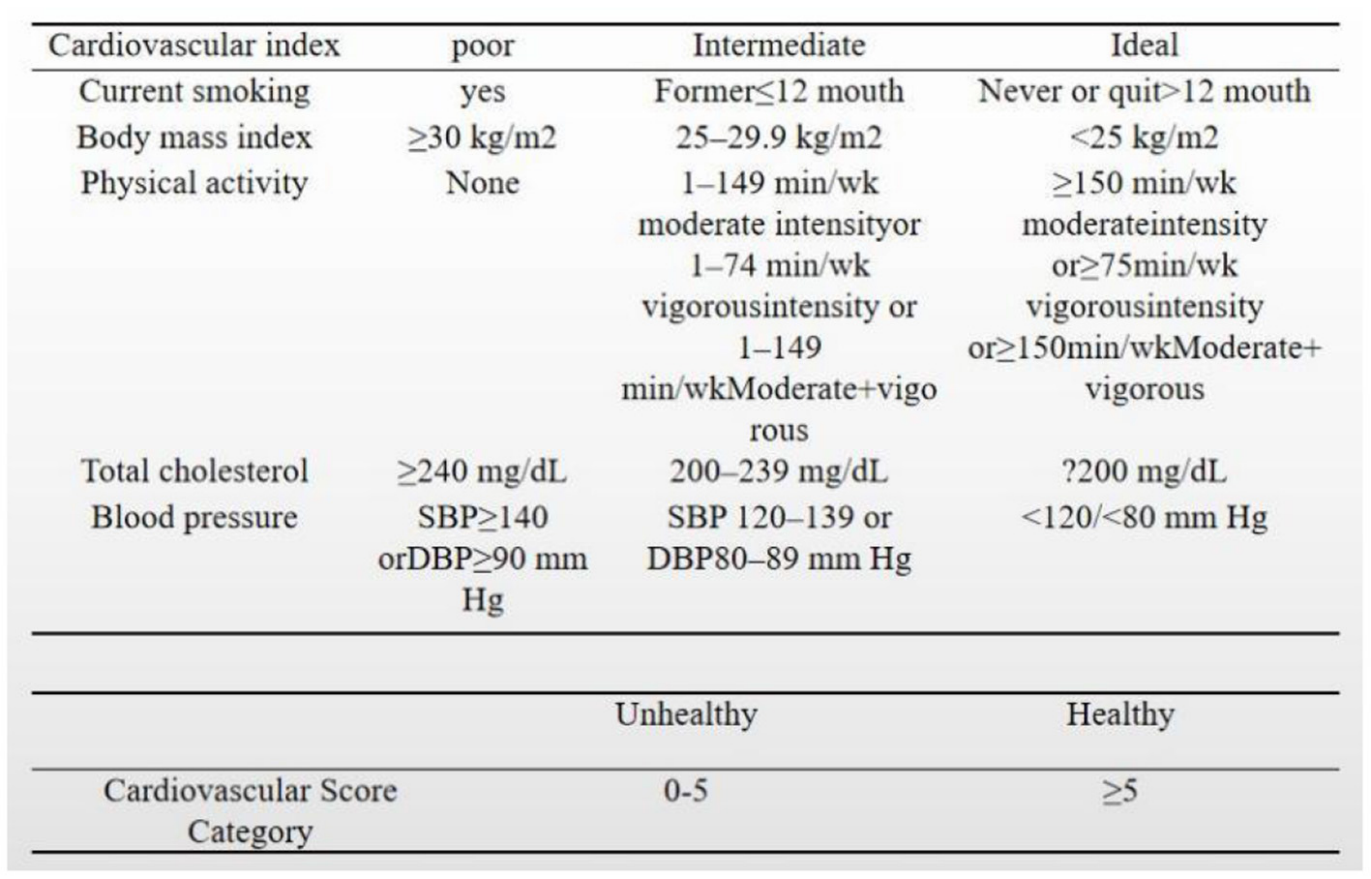
Cardiovascular health definition.

The outcome variable was cardiovascular health, the definition of the latest cardiovascular health is not just for diseases. Health is a more extensive concept, and it should also include the body, psychological and social functions, and other components. Therefore, we consider using cardiovascular health more than using cardiovascular disease alone. It is measured by 7 indicators to measure cardiovascular health. It is measured by 7 indicators (smoking, BMI, physical activity, empty blood sugar, blood pressure, cholesterol, diet) (11). Since there is no dietary data for 19–20 years in the NHANES database, and our study target was on patients with diabetes, we used the remaining five indicators to define cardiovascular health (Figure 1). According to the definition of the AHA (American Heart Association), each index of cardiovascular health is scored as ideal, moderate, or poor. We assigned a value of 0 for poor, 1 for moderate, and 2 for ideal, giving a total score of 0–10. Total scores of ≥5 and 0–5 were used to indicate healthy and unhealthy cardiovascular fitness, respectively.

The race, marital status, and education of the subjects were classified by the data codes in the database. Smoking status, physical activity, blood pressure, BMI, and total cholesterol were all classified as defined in Figure 1. Alcohol drinkers were defined as consuming at least 12 alcohol intake per year. Laboratory data are presented as continuous variables.

### Statistical Analysis

Continuous and categorical variables of baseline data were presented as mean ± standard-deviation values and count and percentage values, respectively. Multivariate logistic regression was applied to metformin use in patients with diabetes to analyze its relationship with cardiovascular health outcomes. The logical regression model has adjusted the age, gender, race, marriage, education level, drinking, and some laboratory data. To further test this relationship, we performed subgroup analyses by age, marital status, and alcohol use, and introduced interaction effects using the Wald test.

All statistical analyses in the article were performed using R software, all tests were two-sided, and the significance cutoff was *P* = 0.05.

## Results

Table 1 lists the baseline data of all subjects according to whether or not they took metformin. This study included 1,355 subjects, and similar numbers of people took or did not take metformin. There were slightly more males than females, and the mean age was around 62 years. The table also lists demographic data such as race and marital status, as well as related laboratory indicators.

**Table 1:** Baseline information.

**Variable**	**No-Metformin**	**Metformin**	***P***
**Total**	749	606	
**Sex**			0.117
Male	397 (53)	347 (57.3)	
Female	352 (47)	259 (42.7)	
**Age**	62 (51.71)	63 (55.71)	0.029
**Age**			0.066
<65	450 (60.1)	334 (55.1)	
≥65	299 (39.9)	272 (44.9)	
**Race**			<0.001
Mexican American	85 (11.3)	92 (15.2)	
Other hispanic	80 (10.7)	62 (10.2)	
Non-hispanic white	264 (35.2)	179 (29.5)	
Non-hispanic black	226 (30.2)	157 (25.9)	
Other Race	94 (12.6)	116 (19.1)	
**Education level**			0.384
<9th grade	71 (9.5)	75 (12.4)	
9–11th grade	99 (13.2)	72 (11.9)	
High school graduate	203 (27.1)	149 (24.6)	
Some college or AA degree	244 (32.6)	196 (32.3)	
College graduate or above	132 (17.6)	114 (18.8)	
**Marital status**			0.032
Married/living with partner	428 (57.1)	386 (63.7)	
Widowed/divorced/separated	232 (31)	167 (27.6)	
Never married	89 (11.9)	53 (8.7)	
**Drinking**			0.243
Yes	672 (89.7)	555 (91.6)	
No	77 (10.3)	51 (8.4)	
Albumin	3.9 (3.7, 4.2)	4 (3.8, 4.2)	<0.001
Haematuria	16 (12, 20)	15 (12, 20)	0.269
Serum creatinine	0.9 (0.7, 1.2)	0.9 (0.7, 1)	<0.001
Glucose	122 (102, 156)	126 (103, 162)	0.257
Blood urea nitrogen	0.4 (0.3, 0.5)	0.4 (0.3, 0.6)	0.824
Total protein	7.1 (6.8, 7.4)	7.1 (6.8, 7.5)	0.78
Uric acid	0.6 (4.6, 6.7)	5.5 (4.6, 6.8)	0.799

**Figure 2:**
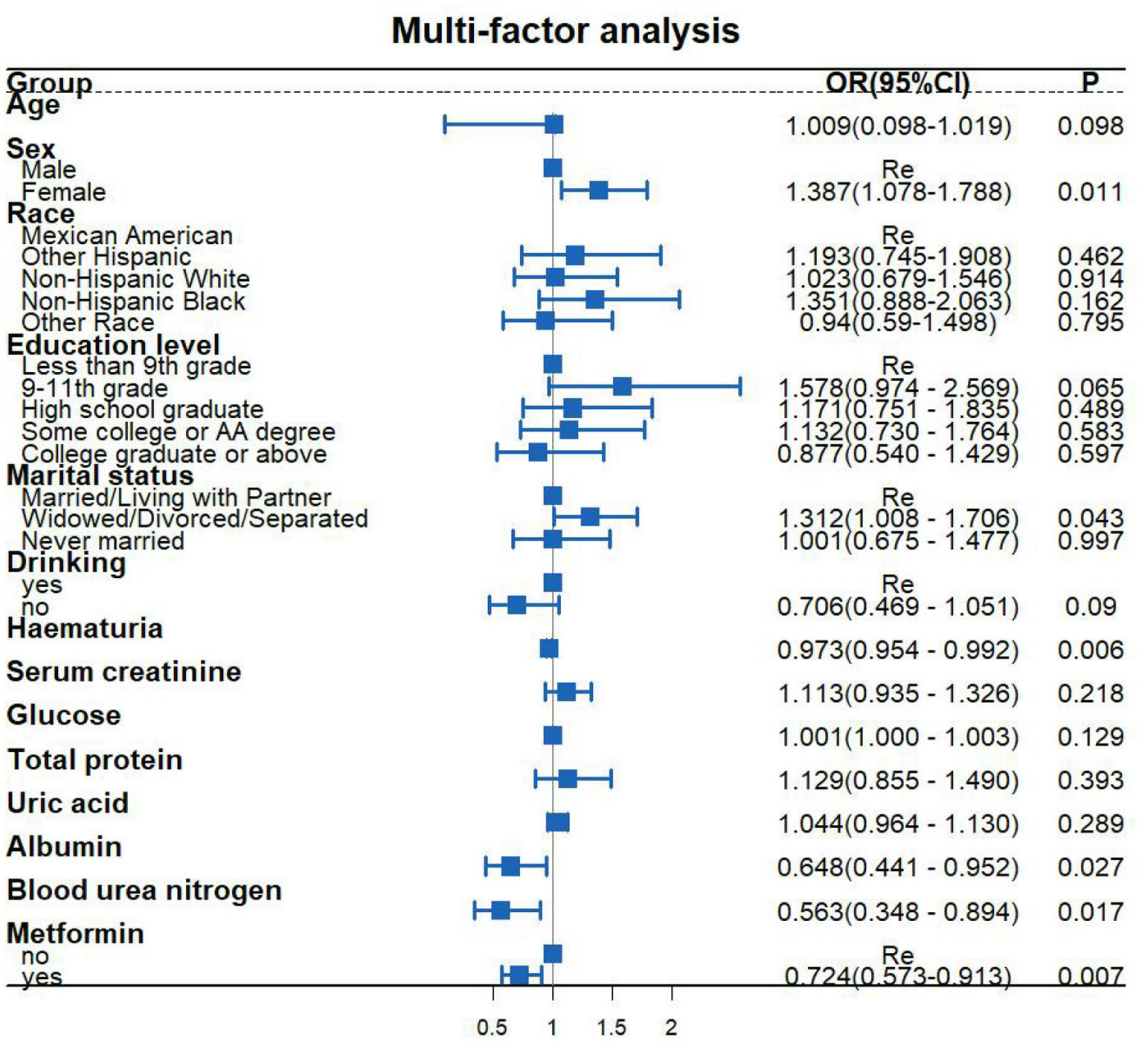
Multi-factor analysis.

Figure 2 is a forest diagram of the relationship between metformin use and cardiovascular disease in patients with diabetes. A logical regression model that adjusts all variables (including age, gender, race, marriage, education level, albumin, hematuria, serum creatinine, glucose, blood urea nitrogen, total protein, uric acid) to determine the effect of metformin use on cardiovascular disease in patients with diabetes. Results show that taking metformin is related to the protection of cardiovascular health in patients (OR: 0.724; 95%CI: 0.573, 0.913; *P* = 0.007).

**Figure 3:**
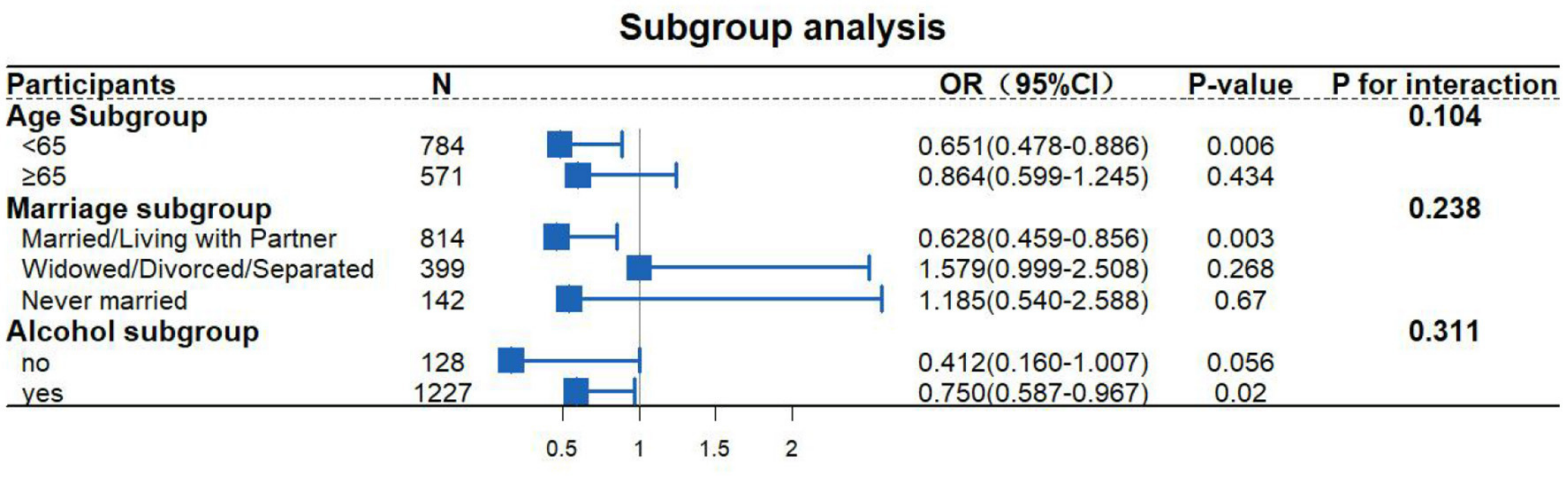
Subgroup analysis.

Figure 3 shows the results of a subgroup analysis that further verified the effect of metformin on cardiovascular health in people with diabetes. After adjusting for all confounding factors, it can be seen that there were significant differences between the young (OR = 0.651, 95% CI = 0.478–0.886, *P* = 0.006), married (OR = 0.628, 95% CI = 0.459–0856, *P* = 0.003), and alcohol-drinking (OR = 0.750, 95% CI = 0.587–0.967, *P* = 0.020) populations.

We also independently analyzed the interactions of metformin with age, marital status, and alcohol consumption, which were all not significant (*P* = 0.104, *P* = 0.238, and *P* = 0.311, respectively).

## Discussion

The study data of patients with diabetes from 2017 to 2020 were extracted from the NHANES database to explore researchers' use of metformin affects their own cardiovascular health. Our results indicated that metformin patients taking metformin are related to better cardiovascular protection, and the results in the subsequent subgroup analyses were more prominent in the younger, married, alcohol-drinking, and highly educated populations.

Studies have found that people with diabetes often also experience cardiovascular events (12, 13). It is therefore of great significance to study the effect of diabetes treatment on cardiovascular events (14). Metformin is the drug of choice for the clinical treatment of patients with diabetes (15). However, recent research findings on the relationship between metformin and cardiovascular events have been controversial (6, 7). The present study found that metformin has a significant protective effect on cardiovascular health. There have been previous studies on metformin use with cardiovascular disease or death as outcomes, but few studies have included cardiovascular health as an outcome. Our study therefore analyzed the relationship between metformin use and cardiovascular disease in patients with diabetes in greater depth.

Several previous prospective randomized controlled trials (16–18) have provided strong evidence for the cardiovascular protection of metformin. However, there is no exact description of the protective mechanism of metformin on the cardiovascular system. In the literature, metformin is considered to reduce the transformation of monocytes to macrophages and the formation of endothelial activation markers (19, 20), both of which are early events in atherosclerosis. Body weight and the fat distribution are also risk factors for cardiovascular health, as found by a study comparing metformin with placebo (21). Metformin can significantly reduce the body weight, and there is also evidence that metformin induces modest changes in blood lipid levels, especially in cholesterol, and triglyceride regulation (22, 23). Long-term maintenance of high blood sugar levels in the body may cause sugars to stick to cellular proteins (24). Metformin promotes a combination of oxidative stress and inflammation, a process called sugar oxidation, which is also responsible for diabetes complications, and metformin neutralizes the intermediates (25) to inhibit the glucose oxidation process, while reducing the occurrence of cardiovascular events (26).

To further demonstrate the protective effect of metformin on cardiovascular events, we performed subgroup analyses by age, marital status, and alcohol use. We found that metformin protects cardiovascular health more significantly in younger populations, mostly because older populations can have greater physiological decline and arterial stiffness, often accompanied by complications such as atherosclerosis and stroke (27, 28), which greatly impact cardiovascular health (29). These may be factors affecting metformin expression in the cardiovascular health of elderly patients with diabetes. Unsatisfactory social relationships can lead to poor habits, in turn leading to the occurrence of psychological and physical diseases (30). A recent prospective study found that loneliness can also have a great impact on cardiovascular health, including in people who are divorced or widowed (31), thereby affecting metformin expression in cardiovascular health. Our subgroup analysis of alcohol use found the relationship to be more meaningful in people who drink alcohol, but because most of the included subjects consumed alcohol, the results for people who did not drink alcohol could be erroneous. Moderate alcohol drinking has positive effects on cardiovascular health (32, 33), and taking metformin has a protective effect on cardiovascular health in people who drink alcohol. It can therefore be speculated that moderate alcohol drinking in patients with diabetes and taking metformin can have complementary effects on cardiovascular health.

Strengths of this study included (1) the subjects being randomly invited to participate in the NHANES, which is a very nationally representative population, and (2) the cardiovascular health scoring system being based on the AHA definition, which ensures the reliability of the study. The study also had some limitations. Because it had a cross-sectional design, we cannot accurately infer the causal relationship between metformin and cardiovascular health. Due to the restrictions of the NHANES database, we cannot analyze the combination of other anti -sugar drugs and metformin drugs. Also, cardiovascular health may be affected by many other factors, and so bias may have been present in the research results.

## Conclusion

This study found that metformin patients taking metformin are related to better cardiovascular protection. This protective effect was more pronounced in people who were younger, married, and drank alcohol. Our findings may therefore further support the use of metformin by people with diabetes.

## Data Availability Statement

Publicly available datasets were analyzed in this study. This data can be found here: (Nhanes) https://www.cdc.gov/nchs/nhanes/index.htm.

## Ethics Statement

Written informed consent was obtained from the individual(s) for the publication of any potentially identifiable images or data included in this article.

## Author Contributions

JL conceptualized the research aims and planned the analyses. CC and SY guided the literature review and wrote the first draft of the paper and the other authors provided comments and approved the final manuscript. XZ and MQ extracted the data from the NHANES database. XZ, SL, NH, LH, and MQ participated in data analysis and interpretation. All authors contributed to the article and approved the submitted version.

## Funding

This study was supported by Guangdong Provincial Key Laboratory of Traditional Chinese Medicine Informatization (2021B1212040007).

## Conflict of Interest

The authors declare that the research was conducted in the absence of any commercial or financial relationships that could be construed as a potential conflict of interest.

## Publisher's Note

All claims expressed in this article are solely those of the authors and do not necessarily represent those of their affiliated organizations, or those of the publisher, the editors and the reviewers. Any product that may be evaluated in this article, or claim that may be made by its manufacturer, is not guaranteed or endorsed by the publisher.

## References

[1] Tucker LA. Dietary fiber and telomere length in 5674 U.S. adults: an NHANES study of biological aging. Nutrients. (2018) 10:400. 10.3390/nu10040400 29570620 PMC5946185

[2] Ma T Huang X Zheng H Huang G Li W Liu X. SFRP2 improves mitochondrial dynamics and mitochondrial biogenesis, oxidative stress, and apoptosis in diabetic cardiomyopathy. Oxid Med Cell Longev. (2021) 2021:9265016. 10.1155/2021/9265016 34790288 PMC8592716

[3] Magzoub R Kheirelseid E Perks C Lewis S. Does metformin improve reproduction outcomes for non-obese, infertile women with polycystic ovary syndrome? Meta-analysis and systematic review. Eur J Obstet Gynecol Reprod Biol. (2022) 271:38–62. 10.1016/j.ejogrb.2022.01.025 35149444

[4] Roth GA Mensah GA Johnson CO Addolorato G Ammirati E Baddour LM. Global burden of cardiovascular diseases and risk factors, 1990-2019: update from the GBD 2019 study. J Am Coll Cardiol. (2020) 76:2982–3021. 10.1016/j.jacc.2020.11.010 33309175 PMC7755038

[5] Raghavan S Vassy JL Ho YL Song RJ Gagnon DR Cho K. Diabetes Mellitus-Related All-Cause and cardiovascular mortality in a national cohort of adults. J Am Heart Assoc. (2019) 8:e11295. 10.1161/JAHA.118.011295 30776949 PMC6405678

[6] Zilov AV Abdelaziz SI AlShammary A Al ZA Amir A Assaad KS. Mechanisms of action of metformin with special reference to cardiovascular protection. Diabetes Metab Res Rev. (2019) 35:e3173. 10.1002/dmrr.3173 31021474 PMC6851752

[7] Griffin SJ Leaver JK Irving GJ. Impact of metformin on cardiovascular disease: a meta-analysis of randomised trials among people with type 2 diabetes. Diabetologia. (2017) 60:1620–9. 10.1007/s00125-017-4337-9 28770324 PMC5552849

[8] Wu WT Li YJ Feng AZ Li L Huang T Xu AD. Data mining in clinical big data: the frequently used databases, steps, and methodological models. Mil Med Res. (2021) 8:44. 10.1186/s40779-021-00338-z 34380547 PMC8356424

[9] Yang J Li Y Liu Q Li L Feng A Wang T. Brief introduction of medical database and data mining technology in big data era. J Evid Based Med. (2020) 13:57–69. 10.1111/jebm.12373 32086994 PMC7065247

[10] Li S Sun W Zhang D. Association of zinc, iron, copper, and selenium intakes with low cognitive performance in older adults: a Cross-Sectional study from national health and nutrition examination survey (NHANES). J Alzheimers Dis. (2019) 72:1145–57. 10.3233/JAD-190263 31683474

[11] Steinberger J Daniels SR Hagberg N Isasi CR Kelly AS Lloyd-Jones D. Cardiovascular health promotion in children: challenges and opportunities for 2020 and beyond: a scientific statement from the american heart association. Circulation. (2016) 134:e236–55. 10.1161/CIR.0000000000000441 27515136 PMC5218582

[12] Wu J Zheng H Liu X Chen P Zhang Y Luo J. Prognostic value of secreted frizzled-related protein 5 in heart failure patients with and without type 2 diabetes mellitus. Circ Heart Fail. (2020) 13:e007054. 10.1161/CIRCHEARTFAILURE.120.007054 32842761

[13] Mai L Wen W Qiu M Liu X Sun L Zheng H. Association between prediabetes and adverse outcomes in heart failure. Diabetes Obes Metab. (2021) 23:2476–83. 10.1111/dom.14490 34227220

[14] Wu L Gunton JE. The changing landscape of pharmacotherapy for diabetes mellitus: a review of cardiovascular outcomes. Int J Mol Sci. (2019) 20:5853. 10.3390/ijms20235853 31766545 PMC6928800

[15] Lee CG Heckman-Stoddard B Dabelea D Gadde KM Ehrmann D Ford L. Effect of metformin and lifestyle interventions on mortality in the diabetes prevention program and diabetes prevention program outcomes study. Diabetes Care. (2021) 44:2775–82. 10.2337/dc21-1046 34697033 PMC8669534

[16] Effect of intensive blood-glucose control with metformin on complications in overweight patients with type 2 diabetes (UKPDS 34). UK Prospective Diabetes Study (UKPDS) Group. Lancet. (1998) 352:854–65. 10.1016/S0140-6736(98)07037-8 9742977

[17] Kooy A de Jager J Lehert P Bets D Wulffele MG Donker AJ. Long-term effects of metformin on metabolism and microvascular and macrovascular disease in patients with type 2 diabetes mellitus. Arch Intern Med. (2009) 169:616–25. 10.1001/archinternmed.2009.20 19307526

[18] Hong J Zhang Y Lai S Lv A Su Q Dong Y. Effects of metformin versus glipizide on cardiovascular outcomes in patients with type 2 diabetes and coronary artery disease. Diabetes Care. (2013) 36:1304–11. 10.2337/dc12-0719 23230096 PMC3631843

[19] De Jager J Kooy A Lehert P Bets D Wulffele MG Teerlink T. Effects of short-term treatment with metformin on markers of endothelial function and inflammatory activity in type 2 diabetes mellitus: a randomized, placebo-controlled trial. J Intern Med. (2005) 257:100–9. 10.1111/j.1365-2796.2004.01420.x 15606381

[20] Yang Q Yuan H Chen M Qu J Wang H Yu B. Metformin ameliorates the progression of atherosclerosis via suppressing macrophage infiltration and inflammatory responses in rabbits. Life Sci. (2018) 198:56–64. 10.1016/j.lfs.2018.02.017 29452166

[21] Lachin JM Christophi CA Edelstein SL Ehrmann DA Hamman RF Kahn SE. Factors associated with diabetes onset during metformin versus placebo therapy in the diabetes prevention program. Diabetes. (2007) 56:1153–9. 10.2337/db06-0918 17395752 PMC2533728

[22] Wulffele MG Kooy A de Zeeuw D Stehouwer CD Gansevoort RT. The effect of metformin on blood pressure, plasma cholesterol and triglycerides in type 2 diabetes mellitus: a systematic review. J Intern Med. (2004) 256:1–14. 10.1111/j.1365-2796.2004.01328.x 15189360

[23] Henning RJ. Type-2 diabetes mellitus and cardiovascular disease. Future Cardiol. (2018) 14:491–509. 10.2217/fca-2018-0045 30409037

[24] Katakami N. Mechanism of development of atherosclerosis and cardiovascular disease in diabetes mellitus. J Atheroscler Thromb. (2018) 25:27–39. 10.5551/jat.RV17014 28966336 PMC5770221

[25] Beisswenger PJ Howell SK Touchette AD Lal S Szwergold BS. Metformin reduces systemic methylglyoxal levels in type 2 diabetes. Diabetes. (1999) 48:198–202. 10.2337/diabetes.48.1.198 9892243

[26] Tessier D Maheux P Khalil A Fulop T. Effects of gliclazide versus metformin on the clinical profile and lipid peroxidation markers in type 2 diabetes. Metabolism. (1999) 48:897–903. 10.1016/S0026-0495(99)90226-3 10421233

[27] Christensen K Doblhammer G Rau R Vaupel JW. Ageing populations: the challenges ahead. Lancet. (2009) 374:1196–208. 10.1016/S0140-6736(09)61460-4 19801098 PMC2810516

[28] Lakatta EG. Arterial and cardiac aging: Major shareholders in cardiovascular disease enterprises: Part III: Cellular and molecular clues to heart and arterial aging. Circulation. (2003) 107:490–7. 10.1161/01.CIR.0000048894.99865.02 12551876

[29] Lakatta EG. Cardiovascular regulatory mechanisms in advanced age. Physiol Rev. (1993) 73:413–67. 10.1152/physrev.1993.73.2.413 8475195

[30] Cacioppo JT Hughes ME Waite LJ Hawkley LC Thisted RA. Loneliness as a specific risk factor for depressive symptoms: cross-sectional and longitudinal analyses. Psychol Aging. (2006) 21:140–51. 10.1037/0882-7974.21.1.140 16594799

[31] Roijers J Sunamura M Utens EM Dulfer K Ter Hoeve N van Geffen M. Marital quality and loneliness as predictors for subjective health status in cardiac rehabilitation patients following percutaneous coronary intervention. Eur J Prev Cardiol. (2016) 23:1245–51. 10.1177/2047487316636259 26912741

[32] Cuesta A Haseeb S Aquistapache F Grosso P Alexander B Hopman W. Alcohol consumption and cardiovascular health: a nationwide survey of Uruguayan cardiologists. Alcohol. (2019) 79:163–9. 10.1016/j.alcohol.2019.02.002 30769023

[33] Hernandez-Hernandez A Gea A Ruiz-Canela M Toledo E Beunza JJ Bes-Rastrollo M. Mediterranean Alcohol-Drinking pattern and the incidence of cardiovascular disease and cardiovascular mortality: the SUN project. Nutrients. (2015) 7:9116–26. 10.3390/nu7115456 26556367 PMC4663584

